# Postmenopausal onset of androgen excess: a diagnostic and therapeutic algorithm based on extensive clinical experience

**DOI:** 10.1007/s40618-023-02297-9

**Published:** 2024-02-13

**Authors:** M. Luque-Ramírez, L. Nattero-Chávez, C. Rodríguez-Rubio Corona, A. E. Ortiz-Flores, A. M. García-Cano, M. Rosillo Coronado, B. Pérez Mies, I. Ruz Caracuel, H. F. Escobar-Morreale

**Affiliations:** 1 Diabetes, Obesity, and Human Reproduction Research Group, Instituto Ramón y Cajal de Investigación Sanitaria (IRYCIS) & Universidad de Alcalá, Centro de Investigación Biomédica en RED (CIBERDEM), Instituto de Salud Carlos III, Madrid, Spain; 2 Department of Endocrinology and Nutrition, Hospital Universitario Ramón y Cajal, Carretera de Colmenar Viejo, KM 9.1, 28034 Madrid, Spain; 3 Department of Gynecology and Obstetrics, Hospital Universitario Ramón y Cajal, Madrid, Spain; 4 Department of Internal Medicine, Hospital Universitario de Torrejón, Torrejón de Ardoz, Madrid, Spain; 5 Department of Biochemistry, Hospital Universitario Ramón y Cajal, Madrid, Spain; 6 Department of Pathology, Hospital Universitario Ramón y Cajal, IRYCIS, CIBERONC, Madrid, Spain

**Keywords:** Adrenal cortex, Androgen, Endocrine tumors, Management of endocrine disease, Ovary

## Abstract

**Purpose:**

Postmenopausal hyperandrogenism is a rare condition that requires identifying those women bearing a life-threatening tumor. We aimed to study diagnostic work-up and management of postmenopausal androgen excess, proposing an algorithm for clinical decision supporting.

**Methods:**

We conducted an observational cross-sectional study and longitudinal follow-up including 51 consecutive menopausal patients reported for hyperandrogenism between 2003 and 2023 to our clinics. We assessed diagnostic testing accuracy and performance by receiver operating characteristic curves, their respective areas under the curve (AUC_ROC_), and 95% confidence intervals (95%CI), for distinguishing between benign and malignant conditions, and androgen excess source.

**Results:**

Most commonly, postmenopausal hyperandrogenism derived from benign conditions such as ovarian hyperthecosis (*n* = 9). However, four (8%) patients had borderline/malignant tumors arising at the ovaries (*n* = 3) or adrenals (*n* = 1). These latter were more likely to develop virilization than those with benign disorders [specificity(95%CI)]: 0.87 (0.69; 0.92)]. Circulating total testosterone [AUC_ROC_(95%CI): 0.899 (0.795; 1.000)] and estradiol [AUC_ROC_(95%CI): 0.912 (0.812; 1.000)] concentrations showed good performances for discriminating between both conditions. Transvaginal-ultrasonography found two out of three potentially malignant ovarian neoplasms, and another was apparent on a pelvic computed tomography scan. An adrenal computed tomography scan also located an androgen-secreting carcinoma.

**Conclusions:**

Clinical or biochemical features of an aggressive androgen-secreting tumor should lead to urgently obtaining a targeted imaging. At first, an abdominal-pelvic CT scan represents the best choice to perceive adrenal malignancy, and may identify aggressive ovarian tumors. When warning signs are lacking, a calm and orderly work-up allows properly addressing the diagnostic challenge of postmenopausal hyperandrogenism.

**Supplementary Information:**

The online version contains supplementary material available at 10.1007/s40618-023-02297-9.

## Introduction

The postmenopausal onset of hyperandrogenism becomes a diagnostic challenge in many cases [1]. The worsening of physical signs of hyperandrogenism early after menopause may be considered part of the physiological aging process, because menopause causes an imbalance between an abrupt decrease in ovarian estrogen synthesis and a gradual decline in androgen concentrations [2]. Thus, in case, a detailed history and a physical examination suggest the presence of previously undiagnosed functional hyperandrogenism during the reproductive years, the polycystic ovary syndrome (PCOS) is a likely diagnosis of that supposed postmenopausal condition [1–3]. However, the abrupt onset of clinical hyperandrogenism or a rapid progression of severe symptoms should prompt the urgent search of potentially life-threatening androgen-secreting tumors [2, 3].

Circulating androgen concentration thresholds for the identification of malignant conditions are controversial, because benign disorders such as ovarian hyperthecosis (OHT) may had severe hyperandrogenemia [1, 4]. Regarding imaging, transvaginal ultrasound (TV-US) is the first-line method for ovarian tumors, although some of them elude sonographic detection [5]. In adrenal conditions, computerized tomography (CT) provides a good discrimination between benign and malignant masses [6]. Lastly, selective adrenal and ovarian venous sampling might contribute to localize the source of androgen excess [7, 8].

Patients with functional conditions diagnosed after menopause such as PCOS or obesity-associated hyperandrogenism can be conservatively managed with changes in lifestyle and/or antiandrogenic therapy. On the contrary, timely surgery is indicated for any suspicious adrenal or ovarian mass. OHT is preferably treated by elective surgery, too [4].

We hereby analyzed our local extensive experience by reviewing the data of the patients consecutively reported for this reason to the Gynecology and Reproductive Endocrinology clinics of a referral Academic Hospital.

## Methods

The study protocol followed the STROBE statement for observational studies [9] and was registered in the Open Science Framework registry (https://osf.io/edrsn/?view_only=64cded2ded6b403cac05b3284c503cb1).

### Subjects

We included all consecutive menopausal individuals reporting to our clinics between 2003 and 2023 because of hyperandrogenism that apparently aroused after menopause (*n* = 51). We retrospectively retrieved data from these patients from their clinical records. We provide exclusion criteria and a detailed definition of each hyperandrogenic condition in those patients who were included in this study as Supplemental Data. Hyperandrogenic disorders were subgrouped into benign or borderline/malignant conditions [10].

### Assays

Assays for sex steroids and gonadotropins, and dynamic endocrine tests are described in the Supplemental Data. Any value of total or free testosterone (T) above our in-house upper limit of normality (ULN) [11, 12] was considered elevated in postmenopausal women. For dehydroepiandrosterone-sulfate (DHEAS), we used the age-adjusted ULN reported by the Quest Diagnostics Nichols Institute using ICLA methodology (https://www.lbm-mg.com/lbm-docs/docsweb/01%20Endo%20Manual.pdf). We expressed circulating androgen values as fold increase in their specific ULN (ULN%) using the formula [(androgen concentration–ULN)*100)/ULN].

### Imaging

Two-dimensional greyscale and spectral Doppler TV-US imaging (4–9 MHz endocavity transducer), and CT and NMR scans were routinely conducted by trained personnel from the Departments of Gynecology and Obstetrics and Radiodiagnostics of our center, respectively.

### NP-59 adrenal scintigraphy

From 3 days before to 5 days after NP-59 intravenous injection, dexamethasone was given to the patients to suppress normal adrenal cholesterol uptake. Each patient received 37 MBq of NP-59. Both anterior and posterior planar images were taken 2, 4, and 7 days after tracer injection.

### Selective adrenal and gonadal venous sampling

Vascular radiologists underwent venous catheterization. They simultaneously obtained blood samples from right adrenal and ovarian veins, left adrenal and ovarian veins, and right brachial vein. We assayed these samples for serum concentrations of total T, androstenedione, DHEAS, estradiol (E_2_), and cortisol.

### Statistical analysis

Descriptive data are shown as mean (standard deviation), median (interquartile range), or counts (%) as appropriate. For continuous variables, we used the Kolmogorov–Smirnov test to check skewness of the distribution. We used nonparametric tests to analyze variables that deviated from normal distribution. Those clinical and biochemical variables of interest were compared between subgroups of patients using Student’s t, ANOVA, Mann–Whitney U or Kruskal–Wallis tests, as needed. Comparisons of discrete variables among study subgroups used χ^2^ or Fisher's exact tests.

We calculated sensitivity (SE), specificity (SP), positive likelihood ratio (PLR), and negative likelihood ratio (NLR), and their 95% confidence intervals (95%CI), of diverse clinical and diagnostic tests for several outcomes.

The overall diagnostic performance of biochemical and radiological quantitative variables for distinguishing between with benign and borderline/malignant conditions was analyzed by receiver-operating characteristic curve (ROC) analyses, their respective areas under the curve (AUC_ROC_), and 95%CI, that also provided optimal thresholds for these variables.

All *P* values were two-sided, and *α* = 0.05 was set as level of statistical significance.

## Results

At first consultation, the mean age and time elapsed from menopause were 64 (9) and 14 (9) yrs., respectively. Clinical and hyperandrogenic features as a function of etiology are shown in Table 1. Hyperandrogenic features at first visit consisted of hirsutism in 37 (73%) women, acne in two (4%) women, and alopecia in 27 (53%) women (Table 1). Ten (20%) women presented with virilization, including clitoromegaly in nine cases and deepening of the voice together with apparently increased muscle mass in another one. Two women with hyperandrogenic features also presented with postmenopausal metrorrhagia. Finally, four women were studied for isolated hyperandrogenemia found during the diagnostic work-up of adrenal incidentalomas in three cases, or during the routine biochemical work-up before bariatric surgery, in another case. Twenty-nine (57%) women had obesity, 37 (73%) had hypertension (of abrupt onset in the case of a woman with an adrenal carcinoma), 25 (49%) had prediabetes or diabetes, and 26 (51%) had dyslipidemia. Fourteen out of 25 (56%) women with prediabetes or type 2 diabetes presented with such a diagnosis before hyperandrogenic complaints onset [median time from dysglycemia to first consultation because of postmenopausal hyperandrogenism of 54 (51) months]. The diagnosis of dysglycemia and hyperandrogenism was simultaneous in six patients (24%), although five of them only showed prediabetes, and the remaining one fulfilled type 2 diabetes diagnostic criteria but without severe hyperglycemia.

**Table 1:** Clinical and hyperandrogenic features as a function of the different sources and etiologies of androgen excess

Source of androgen excess	Etiology, *n* (%)	Age (yrs.)	Time elapsed since menopause (yrs.)	Hyperandrogenic signs at physical examination (%)	Time elapsed since the onset of symptoms (months)
*Ovarian*					
Benign conditions					
	Confirmed ovarian hyperthecosis, 9 (18%)^a^	68 (7)	19 (8)	H (89%), AL (67%), V (22%)	49 (40)
	Gonadotropin-dependent hyperandrogenism with negative imaging during follow-up, 7 (14%)	64 (7)	13 (6)	H (71%), AL (57%)	24 (132)
	Leydig cell hyperplasia, 3 (6%)	65/74/65	13/24/19	H (33%), AL (100%), V (66%)	60/0/36
	Mucinous cystadenoma, 2 (4%)^b^	48/77	15/24	AL (50%), V (50%)	12/24
	Fibrothecoma, 2 (4%)	58/68	6/14	H (50%), AL (50%)	6/-
	Stromal luteoma, 1 (2%)	61	16	H, AL	4
	Leydig cell tumor, 2 (4%)^c^	59/57	17/9	H (100%), AL (50%), V (100%), ↑ libido (50%)	2/6
	Apparently normal ovarian histology, 2 (4%)	71/66	23/21	H (100%), AL (50%), V (50%)	72/36
Borderline/malignant tumors					
	Steroid cell tumor-NOS, 2 (4%)	68/59	15/8	H (50%), AL (100%), V (50%), metrorrhagia (50%)	48/36
	Granulosa cell tumor, 1 (2%)	49	2	H, A, V	4
*Adrenal*					
Benign conditions					
	Nonclassic congenital adrenal hyperplasia, 7 (14%)^d^	68 (19)	18 (15)	H (57%), AL (43%), metrorrhagia (14%), IA (14%)	65 (86)
	ACTH-dependent functional HA, 8 (16%) - PCOS/IH (*n* = 5) - Obesity-associated HA (*n* = 2)	59 (9)	11 (10)	H (88%), A (13%), AL (25%), IA (25%)	63 (128)
	Adrenal adenoma/bilateral macronodular adrenal disease, 2 (4%)^e^	60/60	9/9	H (100%)	12/24
Malignant tumors					
	Adrenal carcinoma, 1 (2%)	65	8	H, AL	3
*Other etiologies*					
	Cushing’s disease, 1 (2%)	50	0	H, AL	24
	GH-secreting pituitary adenoma, 1 (2%)	51	1	H, AL	432
	Analytical cross-reactivity caused by exemestane, 1 (2%)	58	6	IA	0
	Unclear, 1 (2%)	79	29	IA	0

Data are means [SD], medians [interquartile range], raw data when a subgroup includes ≤ 5 women, or counts (%). Four women were studied for isolated hyperandrogenemia found during the diagnostic work-up of adrenal incidentalomas in three cases, or during the routine biochemical work-up before bariatric surgery, in another

*A* acne, *AL* alopecia, *H* hirsutism, *HA* hyperandrogenism, *IA* isolated hyperandrogenemia, *IH* idiopathic hyperandrogenism, *V* virilization (including clitoromegaly in nine cases and deepening of the voice together with apparently increased muscle mass in another one)

^a^One patient had an X polysomy plus 13q deletion

^b^Both cases were associated with ovarian hyperthecosis

^c^One patient carried a pathogenic *BRCA1* mutation

^d^One patient had a confirmed ovarian hyperthecosis and a previously unknown non-classic congenital adrenal hyperplasia

^e^One patient presented simultaneously an adrenal adenoma with aberrant LH/hCG receptor expression and ovarian hyperthecosis

Seven women had suffered from breast carcinoma before seeking assistance for their hyperandrogenic disorder; six of them showed positive hormone receptors. A woman presented with an atypical hyperplasia diagnosed 1 year before receiving help from her hyperandrogenic disorder. One endometrial carcinoma and another atypical endometrial hyperplasia were found after surgery in two women submitted to oophorectomy plus hysterectomy because of an ovarian source of androgen excess.

### Causes and frequencies of androgen excess

The source of androgen excess was ovarian in 29 (57%) women, adrenal in 17 (33%), mixed in two cases (4%), and in one woman was not located (Table 1). The most common cause of postmenopausal hyperandrogenism was histologically confirmed OHT (*n* = 9). Almost 30% of patients presented with functional adrenal hyperandrogenism. Seven (14%) women were diagnosed with non-classic congenital adrenal hyperplasia (NCCAH). Overall, 8 of the 15 women with adrenal functional hyperandrogenism—including 3 diagnosed with NCCAH—described hyperandrogenic features of functional hyperandrogenism during their fertile age. Noticeably, 16 (31%) women presented with some premenopausal hyperandrogenic features, i.e., hirsutism, alopecia, or subfertility and, of them, 15 had a final diagnosis of a benign postmenopausal condition.

Four women (8%) presented with borderline/malignant tumors arising at the ovaries (*n* = 3) or adrenals (*n* = 1). Women with borderline/malignant disorders did not request assistance earlier compared to those with benign ones [20 (42) *vs.* 24 (66) months, respectively]. However, they were more likely to develop virilization than those with benign disorders if those patients without a definitive diagnosis but a non-evident tumor were considered as a whole (Table 2).

**Table 2:** Sensitivity, specificity, positive likelihood ratio and negative likelihood ratio of several clinical, biochemical and radiologic variables for different outcomes

	Outcome	SE, % (95% CI)	SP, % (95% CI)	PLR (95% CI)	NLR (95% CI)
*Androgenic features during fertile age*	Benignant condition				
- *All patients*		83 (59; 96)	97 (84; 100)	27.5 (4.0; 191.6)	0.2 (0.2; 0.5)
- *Only patients with a definitive diagnosis*		36 (21; 53)	75 (19; 99)	1.4 (0.3; 8.3)	0.9 (0.5; 1,6)
*Severe hyperandrogenism (virilisation)*	Potential malignancy				
- *All patients*		50 (7; 93)	87 (69; 92)	2.9 (0.9; 9.4)	0.6 (0.2; 1.6)
- *Only patients with a definitive diagnosis*		6 (1; 20)	20 (3; 56)	0.1 (0.0; 0.3)	4.7 (1.4; 16.3)
*↑ Total testosterone (*≥ *120 ULN%)*	Potential malignancy				
- *All patients*		100 (40; 100)	77 (62; 88)	4.3 (2.6; 7.2)	0.0
- *Only patients with a definitive diagnosis*		100 (40; 100)	72 (55; 85)	3.6 (2.2; 5.9)	0.0
*↑ Calculated free testosterone (*≥ *90 ULN%)*	Potential malignancy				
- *All patients*		100 (40; 100)	63 (46; 77)	2.7 (1.8; 4.0)	0.0
- *Only patients with a definitive diagnosis*		100 (40; 100)	59 (41; 76)	2.5 (1.6; 3.7)	0.0
*Measurable total estradiol*	Potential malignancy				
- *All patients*		100 (40; 100)	74 (58; 86)	3.8 (2.3; 6.3)	0.0
- *Only patients with a definitive diagnosis*		100 (40; 100)	71 (53; 85)	3.4 (2.0; 5.7)	0.0
*↑ Total testosterone* + *measurable total estradiol*	Potential malignancy				
- *All patients*		100 (40; 100)	92 (80; 98)	11.8 (4.6; 30.0)	0.0
- *Only patients with a definitive diagnosis*		100 (40; 100)	90 (76; 97)	9.8 (3.9; 24.7)	0.0
*↑ DHEAS (*> *ULN%)*	Adrenal androgen excess source				
- *All patients*		53 (28; 77)	88 (71; 97)	4.2 (1.5; 11.8)	0.5 (0.3; 0.9)
- *Only patients with a definitive diagnosis*		53 (28; 77)	83 (63; 95)	3.2 (1.2; 8.6)	0.6 (0.3; 1.0)
*Abnormal ovarian finding by TV-US*	Ovarian tumour				
- *All patients*		70 (35; 93)	78 (62; 90)	3.2 (1.6; 6.8)	0.4 (0.2; 1.0)
- *Only patients with a definitive diagnosis*		70 (35; 93)	79 (60; 92)	3.4 (1.5; 7.7)	0.4 (0.1; 1.0)
*Abnormal ovarian finding by TV-US*	Potential malignancy				
- *All patients*		67 (9; 99)	71 (55; 83)	2.3 (0.9; 5.7)	0.5 (0.1; 2.4)
- *Only patients with a definitive diagnosis*		67 (9; 99)	69 (52; 84)	2.2 (0.9; 5.6)	0.5 (0.1; 2.4)
*Gonadotrophin suppression test*	Ovarian androgen excess source				
- *All patients*		92 (62; 100)	67 (9; 99)	2.8 (0.6; 13.8)	0.1 (0.0; 1.0)
- *Only patients with a definitive diagnosis*		92 (62; 100)	67 (9; 99)	2.8 (0.6; 13.8)	0.1 (0.0; 1.0)

We conducted diagnostic test evaluations in all patients (*n* = 51) as a whole and after excluding women without a definitive diagnosis. These latter included those patients with suspected ovarian hyperthecosis or ovarian stromal hyperplasia due to the presence of gonadotropin-dependent clinical and biochemical hyperandrogenism without evidence of androgen-dependent neoplasm during their follow-up nor histopathology confirmation, as well as a patient with an unclear source of androgen excess after 105 months of follow-up

*DHEAS* dehydroepiandrosterone-sulfate, *NLR* negative likelihood ratio, *PLR* positive likelihood ratio, *SE* sensitivity, *SP* specificity, *TV-US* transvaginal-ultrasonography, *ULN* upper limit of normality

### Circulating sex steroids

**Fig. 1:**
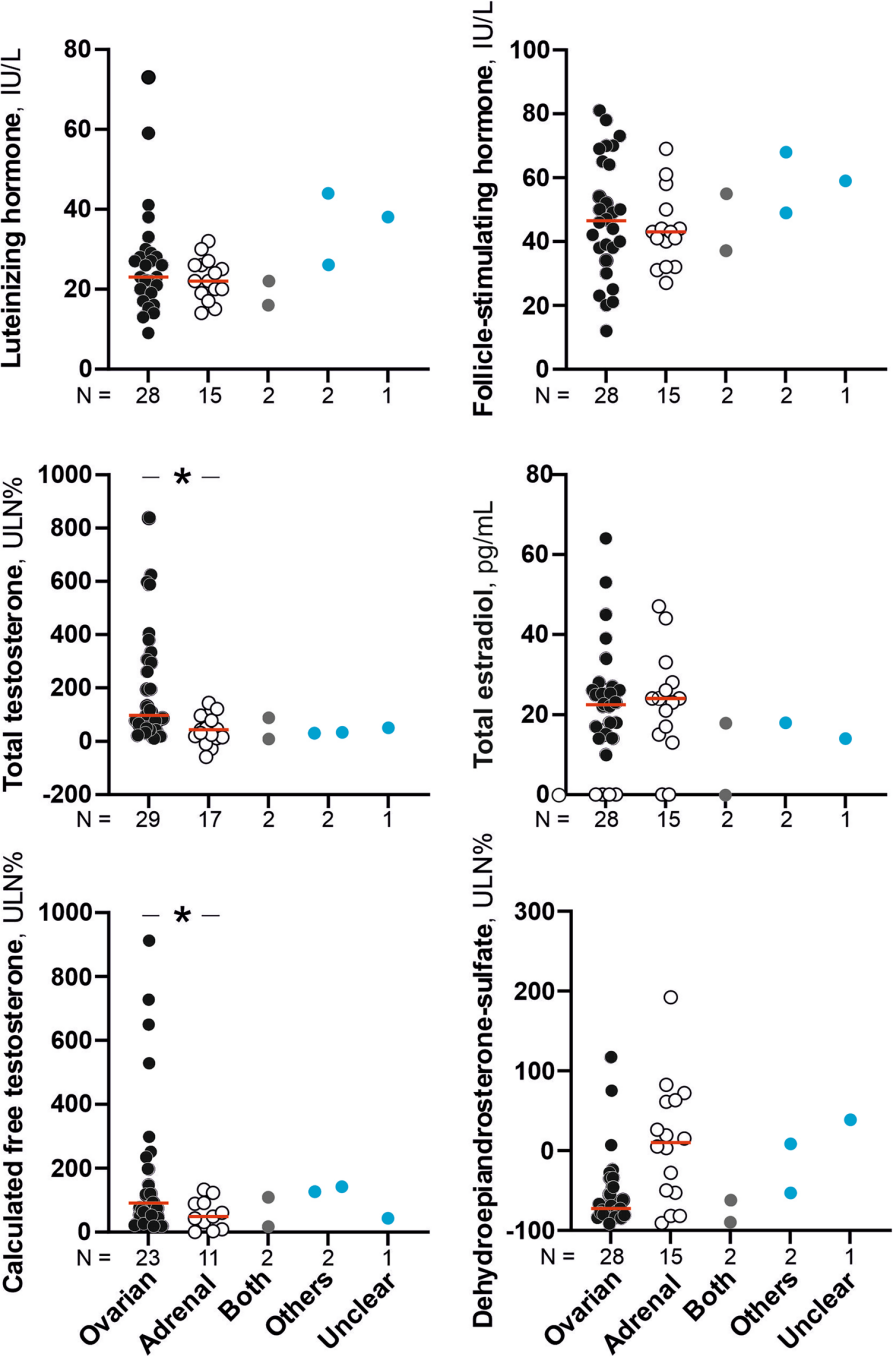
Gonadotrophin and sex steroids concentrations as a function of the source of androgen excess. Individual values of each patient are shown as open dots. The red solid line marks the median of each subgroup of patients. Black circles represent those patients with an ovarian source of androgen excess. White circles represent those patients with an adrenal source of androgen excess. Grey circles represent those patients with both coexisting sources, ovarian and adrenal, of androgen excess. Blue circles represent those patients with an androgen excess from other sources or of an unclear origin. Figures below the X-axis indicates the number of patients in each subgroup. ^*^Statistically significant differences between subgroups

Circulating total and free T were higher in women with ovarian disorders compared to those with adrenal disease, although with overlapping among groups (Fig. 1). Only 13 women showed DHEAS concentrations above the age-related ULN, including 3 women with ovarian androgen excess, and 1 woman presenting with acromegaly. In women with adrenal diseases, both mean DHEAS concentrations were greater and elevated DHEAS concentration were more frequent than in their counterparts showing only gonadal conditions (75% *vs.* 27%, χ^2^:8.770, *P* = 0.005). Of note, 25% of patients with adrenal disorders showed DHEAS concentrations within the normal range. Circulating DHEAS yielded an AUC_ROC_ (95%CI) of 0.674 (0.487; 0.861) for identifying an adrenal disorder.

**Fig. 2:**
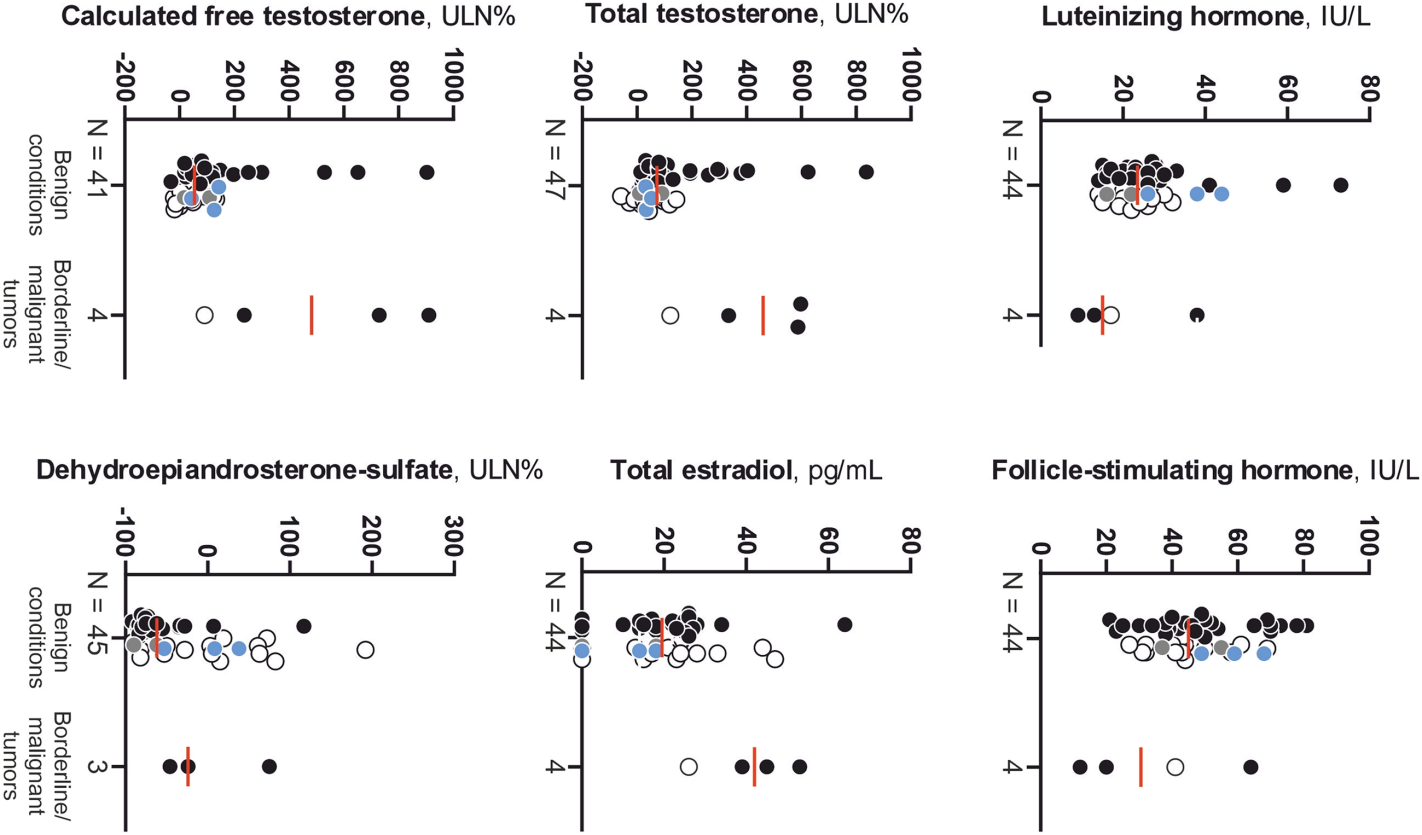
Gonadotropins and sex steroid concentrations in women with benign conditions compared with those of women with borderline/malignant tumors. Black circles represent those patients with an ovarian source of androgen excess. White circles represent those patients with an adrenal source of androgen excess. Grey circles represent those patients with both coexisting sources, ovarian and adrenal, of androgen excess. Blue circles represent those patients with an androgen excess from other sources or of an unclear origin. The red solid line marks the median of each subgroup of women. Figures below X-axis indicates the number of women in each subgroup. ^*^Statistically significant differences between subgroups

**Fig. 3:**
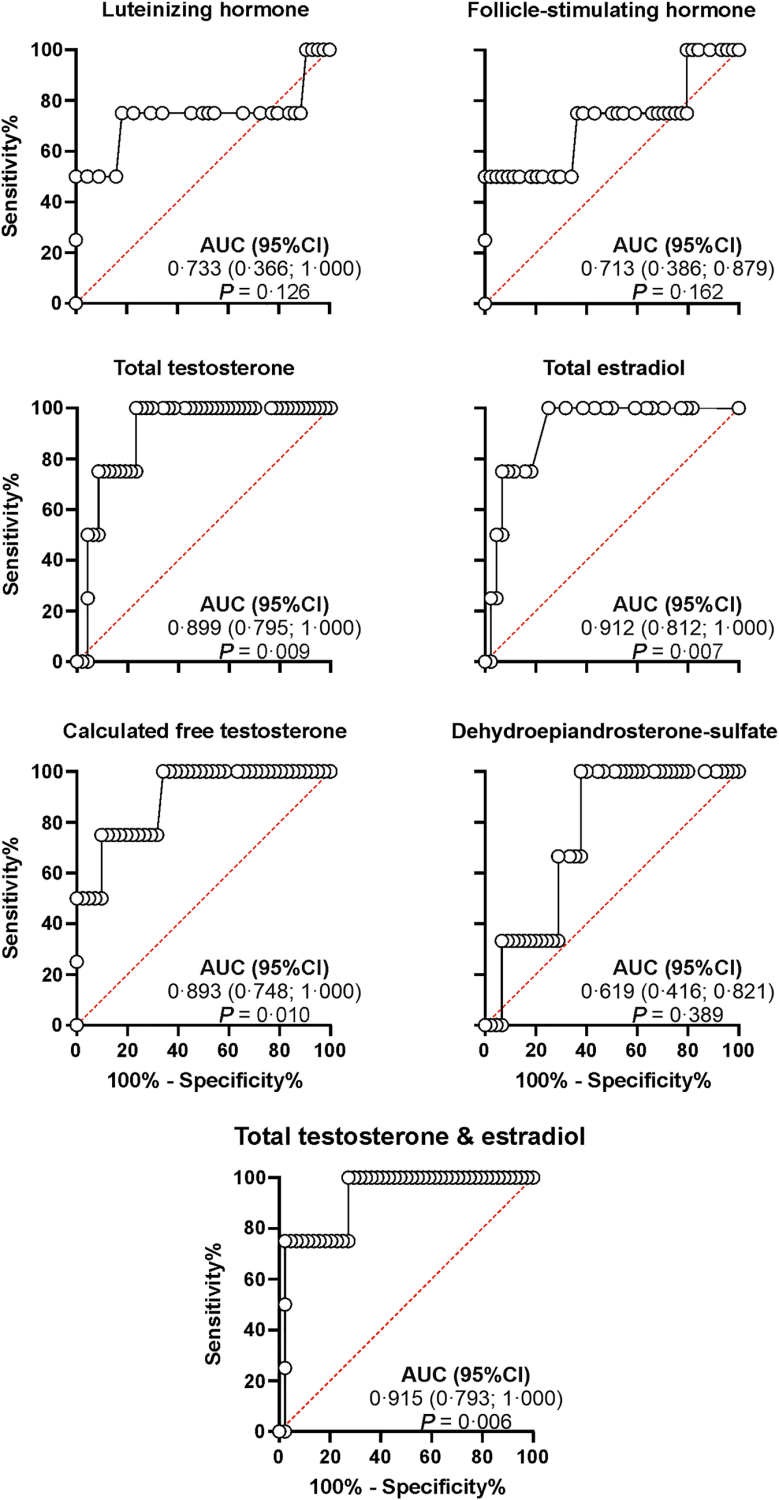
Receiving-operating curves of gonadotropins and sex steroid concentrations for discriminating between benign and borderline/malignant androgen-secreting tumors. *AUC* area under the curve, *CI* confidence interval

The subset of women with borderline/malignant disorders had higher total and free T, and total E_2_ than those with benign conditions (Fig. 2). These sex steroids showed a very good performance (Fig. 3) for discriminating between both conditions with optimal cutoffs of 120 ULN%, 90 ULN%, and 26 pg/mL, for total and free T, and total E_2,_ respectively.

Circulating androstenedione was elevated above in-house ULN in 11 patients, of whom four subjects had NCCAH. We observed a circulating androstenedione ≥ 9 ng/mL in two patients with borderline/malignant conditions (a steroid cell tumor-NOS and an adrenal carcinoma), but was also found in a case with ACTH-dependent functional hyperandrogenism. A very high androstenedione concentration > 80 ng/mL was found in a patient with a breast neoplasm under exemestane administration when using a routine ICLA assay. Suspecting a falsely elevated result, derived from the structural similarity between exemestane and androstenedione, we measured the same sample using a liquid chromatography/tandem mass spectrometry (LC–MS/MS) assay. This gold-standard method [13] confirmed the interference, because actual androstenedione concentrations were 0.4 ng/mL.

All but one patient with confirmed NCCAH had basal 17OHP concentrations > 3 ng/mL, whereas the other one showed a non-stimulated 17OHP concentration of 1.1 ng/mL and of 20 ng/mL after 1–24 ACTH stimulation. Confirmatory molecular genetics analyses were performed in five of them, with two patients carrying severe *CYP21A2* alleles. That patient presenting with an adrenal carcinoma, and two more individuals diagnosed with gonadotrophin-dependent hyperandrogenism, also had increased basal 17OHP concentrations of 3.3, 2.5, and 2.6 ng/mL, respectively.

Finally, three cases presented with endogenous hypercortisolism that originated from a pituitary adenoma, a bilateral macronodular adrenocortical disease, and an adrenal carcinoma.

### Imaging

The findings of pelvic imaging in this cohort of patients with hyperandrogenism are summarized in the Supplementary Table 1. Eleven out of 31 cases (36%) with an ovarian source of androgen excess showed an abnormal ovarian imaging, although such findings were also reported in 31% of patients with adrenal hyperandrogenism. Two out of three confirmed borderline/malignant ovarian neoplasms were found by TV-US, and the other one was apparent on CT. Sonography diagnosed seven out of ten ovarian tumors regardless risk of malignancy.

Adrenal imaging identified three cases with non-functional adrenal hyperandrogenism including the patient with an adrenal carcinoma. However, it also revealed unilateral or bilateral non-functioning adenomas in 5 patients with an ovarian source of androgen excess, and in 8 (53%) of the 15 individuals diagnosed with functional adrenal hyperandrogenism.

NP-59 scintigraphy was positive in the patient with the functioning adrenal adenoma expressing aberrant receptors, and negative in the three cases with positive adrenal CT and/or NMR scans but final diagnoses of ovarian conditions.

### Dynamic endocrine tests

Dynamic functional tests were conducted in 28 patients (Supplementary Table 2). When considering as a whole those patients who received a definitive histopathologic diagnosis, a decrease ≥ 40% in total T after gonadotrophin-suppression by gonadotropin-releasing hormone (GnRH) analogues showed a good diagnostic performance for the identification of an ovarian source of androgen excess [AUC_ROC_ (95%CI) 0.911 (0.778; 1.000)]. Liddle’s tests were conducted in eight patients. Seven out of them—five of those who failed to suppress total T after triptorelin administration—showed decreases in total T levels that were > 40% and received a diagnosis of functional ACTH-dependent hyperandrogenism.

### Selective adrenal and ovarian venous sampling

Five patients were submitted to selective venous sampling, including one who showed ovarian and adrenal lesions in imaging and another patient who had an adnexal cyst but did not suppress circulating total T levels after triptorelin. Being OHT a much more frequent disorder compared with the very rare virilizing adrenal adenoma [14], selective venous sampling was performed in three patients who showed adrenal imaging suggestive of adrenal adenomas despite not showing adnexal findings.

Although adrenal–peripheral venous cortisol ratios indicated unsuccessful right adrenal vein catheterization during all procedures, gonadal–peripheral and left adrenal–peripheral total T ratios correctly identified the source of androgen excess in three patients with an unilateral Leydig cell tumor, an ovarian mucinous cystadenoma associated with OHT, and the adrenal adenoma that showed aberrant receptor expression on histopathology, respectively. All of them showed adrenal–peripheral or gonadal–peripheral total T gradients > 9 that were in agreement with the source of androgen excess as confirmed by surgery and pathology.

Another patient with gonadotrophin-dependent androgen excess, normal adnexal imaging, and a 7 mm adrenal adenoma presented with 2.0 right ovary-peripheral total T gradient, 0.8 left ovary-peripheral total T gradient, and 7.1 left adrenal–peripheral total T gradient. She refused adrenal surgery, and hence, we could not reach a definitive diagnosis in this particular case.

### Management and follow-up

Surgery was the first choice for the management in 22 out of 30 patients with an ovarian source of androgen excess. Bilateral oophorectomies were performed by laparoscopic procedures in 20 cases and by open laparotomy in the other 2. Gynecologic surgeons performed concomitant hysterectomies in five patients and, in three cases presenting with borderline/malignant tumors, hysterectomy plus omentectomy, and peritoneal lavage for staging were conducted at a second stage. These three tumors were limited to one ovary (capsule intact), and did not show malignant cells in peritoneal washings.

In a patient with a final diagnosis of mucinous cystadenoma plus OHT, pelvic adherences—derived from an earlier hysterectomy—prevented the completion of bilateral gonadal resection; hyperandrogenism persisted after surgery being controlled by a GnRH analogue. On the contrary, bilateral oophorectomy solved hyperandrogenemia and improved clinical hyperandrogenism in all cases. Of the 22 patients submitted to ovarian surgery, 21 were alive and relapse of hyperandrogenism was not observed after 79 (47) months of follow-up. The other 1 died of a non-related cause after 212 months of follow-up.

Eight patients with a suspected ovarian cause refused surgery or preferred “wait and see”. All of them are still on active surveillance and neither worsening of hyperandrogenemia nor development of a potentially malignant lesion has been observed after 41 (17) months of follow-up.

Unilateral adrenalectomies were conducted in three patients with disparate results. Debulking surgery, chemotherapy, and mitotane was the only option in that patient with an adrenal carcinoma, since this tumor invaded renal vein and had lung metastases at presentation. The patient died of disseminated disease 21 months after her first visit. While this surgical procedure restored normonandrogenemia in a patient with a final diagnosis of bilateral macronodular adrenocortical disease, without apparent relapse after 59 months of follow-up, clinical and biochemical hyperandrogenism persisted in that presenting with an androgen-secreting adrenal adenoma showing aberrant LH/hCG receptors. The patient underwent bilateral oophorectomy in a second step, which effectively confirmed an OHT, and solved androgen excess.

Six patients diagnosed of NCCAH after menopause only needed dermo-cosmetic measures, added to topical minoxidil in one case, and antiandrogenic therapy with spironolactone in another. A patient with both OHT and NCCAH did not require therapy after bilateral oophorectomy. The eight cases with ACTH-dependent functional hyperandrogenism are on active surveillance and were treated with dermo-cosmetic measures (four cases), oral low-dose prednisone treatment (one case), glucagon-like peptide 1 receptor agonists (one case), bariatric surgery (one case), or spironolactone (one case). None of them developed an apparent potentially malignant tumor after 57 (26) months of follow-up.

Close surveillance remains active for the only individual in whom the source of androgen excess continued to be unclear despite all our diagnostic efforts. Such a source has not surfaced after 105 months of follow-up.

## Discussion

Postmenopausal hyperandrogenism constitutes a rare condition that requires considerable expertise to quickly identify the subset of women bearing an aggressive life-threatening tumor. Aside from this essential concern, postmenopausal hyperandrogenism appears to associate a substantial health burden in the form of hormone-sensitive cancer [15]. Prospective studies associated increased circulating T concentrations with hormone receptor positive breast cancer in menopause [16], and such an association might extend even to tumors lacking expression of hormone receptors [17]. Hyperandrogenism is also a risk factor for endometrial carcinoma during childbearing age, and the same association is likely to apply after menopause [18], as suggested for patients with OHT [19]. Obesity and insulin resistance—common features in postmenopausal patients with functional hyperandrogenism [20]—may drive substantial cardiometabolic comorbidity as well [21]. Hyperandrogenism favors abdominal adiposity and visceral fat depots resulting in insulin resistance [22]. Compensatory hyperinsulinism contributes to ovarian and adrenal hyperandrogenism by acting synergically with LH to stimulate ovarian theca and by facilitating adrenocorticotropin-stimulated androgen secretion [22]. Thus, long-term exposition to a hyperandrogenic milieu must be a matter of concern even if an apparent benign condition is the underlying cause.

**Fig. 4:**
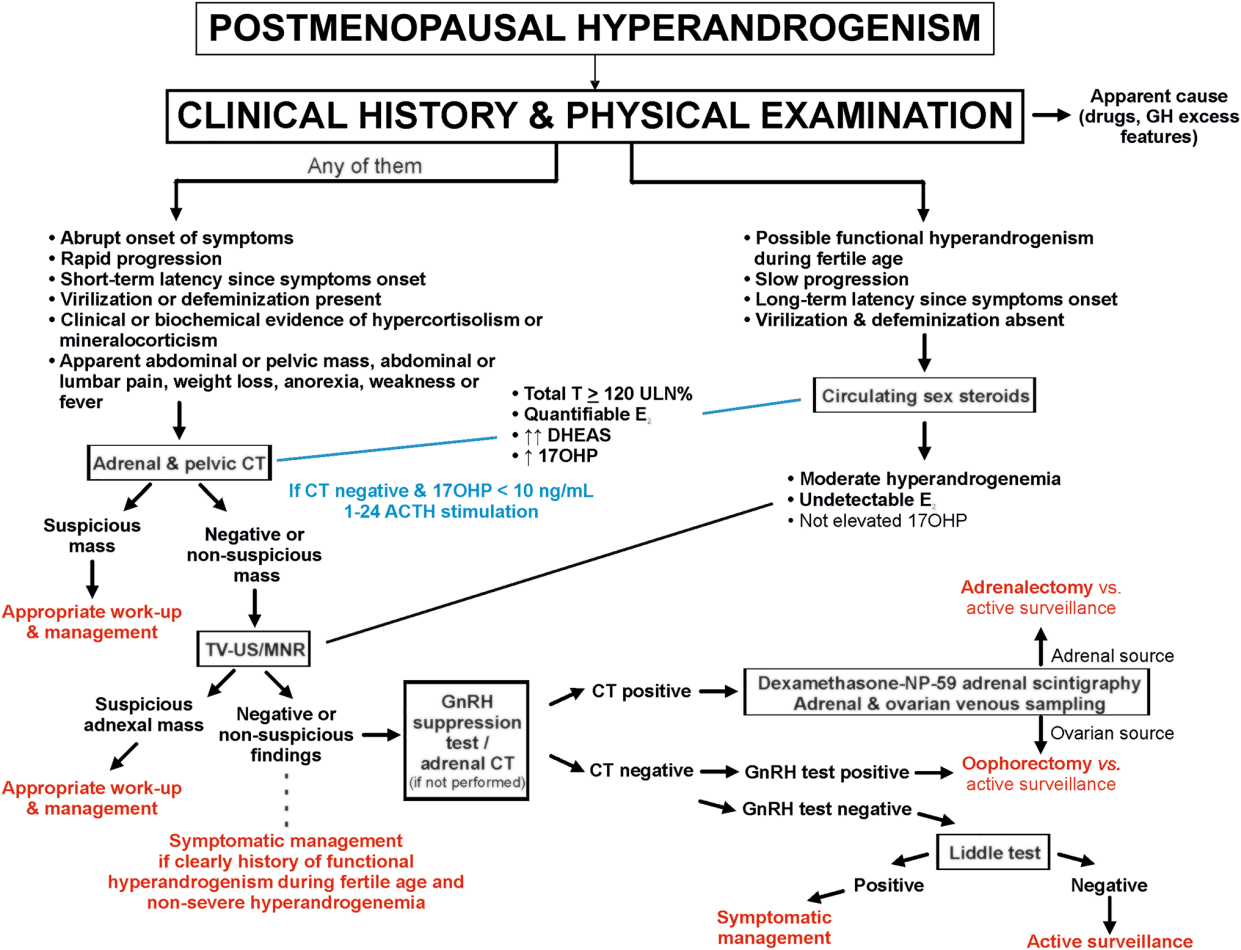
Diagnostic work-up and management of women with onset hyperandrogenism after menopause

Our present data, by providing significant insights into a large series of patients presenting with these conditions, allowed us to propose an orderly diagnostic algorithm (Fig. 4). The first and more important step when facing a patient with postmenopausal hyperandrogenism is to obtain a detailed clinical history and a complete physical examination. Clinical presentation is the key for promptly ruling-out conditions such as virilizing adrenal carcinomas. While borderline/malignant androgen-secreting ovarian tumors may have an indolent course for a long time [23], adrenal carcinomas usually present with a short average duration of symptoms before diagnosis [24, 25]. The presence of clinical or biochemical features suggestive of hypercortisolism may also prompt to discard a co-secreting adrenocortical carcinoma, although rare ovarian steroid cell tumors may have hypercortisolemia [26]. Likewise, severe hyperandrogenic symptoms at physical examination such as virilization are rarely absent in women with potentially malignant hyperandrogenic disorders, yet such symptoms may occur less frequently in functional causes of hyperandrogenism [23]. Recent onset metabolic disturbances derived from severe insulin resistance may be another warning feature in some cases of malignant secreting tumors [27, 28]. However, functional disorders with circulating androgens ranging from mild [21] to severe hyperandrogenemia [29] may present with clinically evident insulin resistance too.

Therefore, when faced in front of any of these clinical indicators of warning, androgen measurements—if not available at this point—ought to never cause any delay in urgently obtaining a targeted imaging (Fig. 4). At first, an abdominal and pelvic CT represents the wisest choice; this imaging technique is not only the best to perceive malignancy in a putative adrenal mass, but also the inclusion of lower abdomen and pelvic regions may identify aggressive ovarian tumors, or even the very unusual but disastrous cause of virilism represented by Krukenberg tumors. If the CT yields negative or non-suspicious adrenal findings, TV-US is an effective method for the detection of virilizing ovarian tumors [5], although NMR may outweigh sonography accuracy in some cases [23].

The absence of any of these alarm signs permits a calm and orderly work-up in which determinations of sex hormones are the first step (Fig. 4). When severe hyperandrogenemia is lacking, a functional form of hyperandrogenism is the most likely etiology for this particular clinical picture. At this point, 8 out of 11 patients of our series had a final diagnosis of NCCAH, of possible pre-existing PCOS, or of obesity-associated functional hyperandrogenism. Noteworthy is the large prevalence of previously unknown diagnoses of NCCAH in our women. Besides the prone Caucasian ethnic background of Spaniards, the relative mild symptoms that characterize some patients with NCCAH, together with the usual worsening of hyperandrogenic features after cessation of ovarian estrogen secretion during menopause, may explain such late diagnoses [2, 30]. Anyhow, it does not preclude considering genetic counselling to the women’s offspring, since carriers of severe alleles are frequent.

Nonetheless, TV-US and/or dynamic studies may be still advisable in these low-risk patients because: i) fibrothecoma, gonadotropin-dependent hyperandrogenism, and an androgen-secreting adrenal adenoma were among the final diagnoses of some of these individuals; and ii) although exceptionally, supranormal 17OHP levels may arise from intratumoral 21-hydroxylase deficiency in some ovarian neoplasms [31].

Regarding circulating androgen concentrations, total T cutoffs for distinguishing between benignant and borderline/malignant conditions are assay-dependent, with red-flag thresholds ranging from 140 to 150 ng/dL for radioimmunoassays [15] to lower concentrations when LC/MS–MS assays are used [32]. We hereby report T thresholds as percentage above ULN to provide an assay-independent advice for clinical practice. In agreement with previous reports, we observed considerable overlapping between the androgen concentrations in some functional conditions and those of androgen-secreting tumors, yet a total T level ≥ 120 ULN% correctly identified all women with a potentially malignant condition. Its diagnostic performance was mildly improved when circulating total E_2_ concentrations were added to the model. Nonetheless, we did not observe any role of DHEAS in discriminating between benignant and malignant conditions. DHEAS concentrations above 6,000–7,000 ng/mL are suggested as a warning threshold for adrenal carcinoma, even though having a limited predictive value [1]. The finding of a very large DHEAS concentration in a woman with an alarming clinical history points to an adrenal carcinoma as the cause of hyperandrogenism, yet such concentrations become a very poor marker of malignancy in asymptomatic women with isolated hyperandrogenemia [33]. Normal DHEAS concentrations never serve to rule out an adrenal carcinoma in case the clinical history and physical examination suggest this possibility.

In the case that previous steps in the diagnostic algorithm failed to provide a presumptive diagnosis and appropriate management, dynamic functional testing may guide decision-making. When GnRH analogues result into suppression of serum T levels, the most likely etiologies, in order of frequency, were OHT, a gonadotrophin-responsive ovarian tumor, or very rarely, an androgen-secreting adrenal adenoma or macronodular adrenal disease with aberrant response to gonadotrophins. If adrenal imaging is normal, laparoscopic bilateral oophorectomy is usually a safe procedure that allows histological diagnostic confirmation while resulting into the complete resolution of hyperandrogenemia in most cases. In patients in whom surgery is contraindicated because of severe comorbidities, or simply refused, active surveillance and medical therapy is a valid alternative.

In the patients with gonadotrophin-dependent androgen excess showing apparently benign adrenal lesions on adrenal imaging, two approaches should be considered depending on local availability and experience. Adrenal scintigraphy may detect autonomous adrenal lesions that, in this context, might lead to an elective adrenalectomy. If there is no apparent NP59 uptake by an adrenal lesion, the etiology is most likely ovarian and bilateral oophorectomy should be advised.

Alternatively, and only if experienced professionals are available, combined adrenal and gonadal venous sampling may be useful to determine androgen excess source. In our local experience, this technique was able to identify correctly the source of hyperandrogenemia in four out of five patients that showed misleading biochemical and radiologic findings, although right adrenal vein catheterization was unsuccessful in all procedures, in agreement with others’ experience [34]. Finally, Liddle´s test served to characterize ACTH-dependent functional hyperandrogenism, in most cases associated to obesity, which is potentially treatable with low-dose glucocorticoids if necessary.

Obviously, our approach may be limited by the relatively small sample size in a rare condition, even though being derived from the largest series of women with postmenopausal hyperandrogenism reported to date. Nevertheless, our systematic approach can be generalized to other populations independently of their ethnic backgrounds, since this suggested diagnostic work-up reasonably cover all plausible etiologies, favoring an adequate diagnosis and correct management.

Hyperandrogenism after menopause is a rare condition that constitutes a challenge even for very experienced physicians. Some causes of postmenopausal virilization are life-threatening tumors that require rapid identification and immediate treatment. Furthermore, although overall survival does not appear to be committed in these patients when applying an appropriate diagnostic work-up and management, available evidence suggest that individuals carrying these hyperandrogenic conditions may suffer from cardiometabolic morbidity and hormone-sensitive cancer. An accurate clinical history and physical examination are the basis for a correct diagnosis and appropriate management. Based on our 2-decade extensive clinical experience, we hereby proposed an orderly work-up algorithm that may facilitate the routine diagnosis and management of these patients.

### Supplementary Information

Below is the link to the electronic supplementary material.

Supplementary file1 (DOCX 30 KB)

## Data Availability

All data sets generated during and/or analyzed during the current study are not publicly available but are available from the corresponding author on reasonable request. Data are also available to the editors of the journal for review or query upon request.

## Abbreviations

ACTH: Corticotrophin
CT: Computerized tomography
DHEAS: Dehydroepiandrosterone-sulfate
E_2_: Estradiol
GnRH: Gonadotropin-releasing hormone
17OHP: 17-Hydroxyprogesterone
ULN: Upper limit of normality
MNR: Magnetic nuclear resonance
T: Testosterone
TV-US: Transvaginal ultrasound
