# Supplementary material for: Postmenopausal onset of androgen excess: a diagnostic and therapeutic algorithm based on extensive clinical experience
Source: J Endocrinol Invest. 2024 Feb 13;47(8):2007–20. doi: 10.1007/s40618-023-02297-9 (PMC11266381; doi:10.1007/s40618-023-02297-9)
Supplement: Supplementary file 1 — Supplementary file1 (DOCX 30 KB) [file 40618_2023_2297_MOESM1_ESM.docx]

**TABLE OF CONTENTS**

Exclusion criteria…………………………………………………………………………………... 2

Case definition……………………………………………………………………………………... 2

Follow-up…………………………………………………………………………………………... 2

Assays……………………………………………………………………………………………… 2

Dynamic endocrine tests…………………………………………………………………………… 3

**Supplementary Table 1.**………………………………………………………………………… 4

**Supplementary Table 2**.………………………………………………………………………….. 5

References…………………………………………………………………………………………. 6

**Exclusion criteria**

Exclusion criteria were:

i) Patients known to have a diagnosis of a hyperandrogenic condition in their premenopausal ages, except those in whom a different disorder developed after menopause.

ii) Subjects lacking a minimum set of available data that included age and hyperandrogenic features at diagnosis, circulating androgens, and imaging studies.

iii) A minimum period of 12 months of follow-up after their initial evaluation, needed to address the possible aggressive malignant behavior of any adrenal or ovarian tumor.

**Case definition**

i) Menopausal status was stated in any of the following situations: a diagnosis of physiological menopause, confirmed by at least 12-months of amenorrhea; isurgical menopause (i.e., bilateral oophorectomy); or other causes of verified menopause such as post-chemotherapy or primary ovarian failure.

ii) Previously undiagnosed premenopausal functional hyperandrogenism disorder – such as PCOS or idiopathic hyperandrogenism – in a menopausal woman required the presence of a history of clinical hyperandrogenism that persists after menopause and/or history suggestive of ovulatory dysfunction during their reproductive ages, together with exclusion of other causes of androgen excess.

iii) NCCAH due to 21-hydroxylase deficiency was diagnosed when circulating 17OHP concentration were above 10 ng/ml either basally or after cosyntropin stimulation.

iv) Obesity-associated adrenal functional hyperandrogenism required the demonstration of ACTH-dependent clinical and biochemical hyperandrogenism with an onset after menopause after exclusion of other causes of hyperandrogenism, in the absence of a history of menstrual dysfunction or hyperandrogenism during fertile age.

v) Cushing’s syndrome required the demonstration of autonomous endogenous hypercortisolism as long as pseudoCushing was properly ruled out.

vi) Acromegaly was diagnosed after demonstration of autonomous excess secretion of growth hormone.

vii) Ovarian stromal hyperplasia, OHT, and ovarian or adrenal-secreting tumors required confirmation by histopathology and resolution of clinical and biochemical hyperandrogenism after surgical removal of the ovaries or the adrenal mass.

viii) Suspected OHT or ovarian stromal hyperplasia required proven gonadotropin-dependent clinical and biochemical hyperandrogenism without evidence of androgen-dependent neoplasm during follow-up.

ix) Iatrogenic hyperandrogenism required the unnoticed or self-administration of androgenic or anabolic drugs.

**Follow-up**

We defined the duration of follow-up for patients as the elapsed time from their initial consultation due to postmenopausal hyperandrogenism to the last electronic clinical record entry. In the case of death, we retrieve its cause as recorded in the patient’s clinical record.

**Assays**

Up to February 2020, serum total testosterone (T) samples were assayed in duplicate using a direct unextracted RIA (Spectria^®^ Testosterone RIA, Orion Diagnostica Oy, Espoo, Finland) with analytical and functional detection limits < 2.9 and 8.7 ng/dL (0.1 and 0.3 nmol/L), respectively. Intraassay coefficient of variation (CV) was < 9% for concentrations ≥ 37.5 ng/dL (1.3 nmol/L), and interassay CVs were 11.6% at 95 ng/dL (3.3 nmol/L) and <10% for concentrations ≥ 141 ng/dL (4.9 nmol/L). The cross-reactivity was < 2.7% for all endogenous steroids. This particular assay was validated against several liquid chromatography-tandem mass spectrometry assays using samples from healthy prepubertal and pubertal girls and boys.^1^ Since 2020, we used an immunochemiluminescence assay (ICLA) [ADVIA Centaur ^®^ Testosterone II Assay (TSTII), Siemens Healthcare Diagnostics Ltd., Frimley, Camberley, United Kingdom]. This immunoassay passed successfully CDC HoSt standardization from August 2016 to date. The limits of detection and quantification of this method were 4.9 and 6.9 ng/dL (0.17 and 0.24 nmol/L), respectively. Intraassay CV was <10% for concentrations > 0.37 nmol/L, and interassay CV was <10% for concentrations > (10.6 ng/dL) 0.92 nmol/L. The cross-reactivity was <1.5% for all endogenous steroids. Calculated free T concentrations derived from total T and SHBG ^2^.

Up to 2020, androstenedione and dehydroepiandrosterone-sulfate (DHEAS) were measured using an automated ICLA (IMMULITE 2000, Siemens Healthcare Sector, Erlangen, Germany) with lower limits of quantification (LLOQ) of 2.9 ng/mL (10.1 nmol/L) for androstenedione and 150 ng/mL (0.04 μmol/L) for DHEAS. The mean intraassay and interassay CVs were < 10% for all these assays. Since 2020, our local laboratory used the Liaison XL Diasorin assay for androstenedione and the Advia Centaur XP Siemens assay for DHEAS. Their LLOQ were 0.24 ng/mL (0.84 nmol/L and 150 ng/mL (0.04 μmol/L), respectively, and intraassay and interassay CVs were < 10% for both methods.

Up to August 2021, our routine lab assayed total estradiol (E_2)_ by an automated ICLA method (Architect^®^ Estradiol, Abbot Ireland diagnostics Division, Lisnamuck, Longford, Co. Longford, Ireland) with a LLOQ < 10 pg/mL (37 pmol/L), a functional sensitivity ≤ 25 pg/mL (92 pmol/L), and mean intraassay and interassay CVs <10%. Since September 2021, E_2_ started to be measured by another automated ICLA platform (Alinity i Estradiol, Abbot Ireland, Diagnostics Division, Lisnamuck Lonford, Ireland) with a functional sensitivity of 24 pg/mL (88 pmol/L), mean intraassay and interassay CVs < 8% with mean concentrations below 188 pg/mL (690 pmol/L).

Up to August 2021, luteinizing hormone (LH) and follicle-stimulating hormone (FSH) were measured in a single assay using an automated ICLA method (Architect^®^ FSH, Architect^®^ LH, Abbot Ireland diagnostics Division, Lisnamuck, Longford, Co. Longford, Ireland) with LLOQ of 0·1 IU/l. and mean intraassay and interassay CVs < 10% for both assays. Since September 2021, LH and FSH are measure by another automated ICLA platform (Alinity i LH & FSH, Abbot Ireland, Diagnostics Division, Lisnamuck Lonford, Ireland) with a functional sensitivity of 0.12 IU/L, mean intraassay and interassay CVs <5%.

**Dynamic endocrine tests**

i) Gonadotropin-releasing hormone (GnRH) analogue suppression test: we obtained blood samples for measuring luteinizing hormone (LH), follicle-stimulating hormone (FSH), total estradiol (E_2)_, total T, androstenedione, and DHEAS before and after 21 days of a single intramuscularly injection of 3.75 mg of triptorelin.

ii) Liddle’s tests were performed by administration of 0.5 mg of oral dexamethasone every six hours for two days. Blood samples were obtained before and two hours after the last dose and assayed for serum cortisol, total T, androstenedione, and DHEAS.

**Supplementary Table 1.** Abnormal imaging findings in women with an ovarian source of androgen excess.

| **TV-US** | **Other imaging techniques** | **Final diagnosis** |
| --- | --- | --- |
| Unilateral hyperechoic simple ovarian cyst (216 * 155 mm) | Simple ovarian cyst  (20 * 14 * 22 cm) (CT) | Mucinous cystadenoma plus stromal luteinization |
| Unilateral ovarian cyst (49 * 30 cm) | Simple ovarian cyst (45 mm) (CT) | Mucinous cystadenoma plus OHT |
| Hyperechoic formation in uterine cavity (26 x 37 mm) with ↑ vascularization plus hypoechoic left ovarian cyst (22 x 19 mm). Endometrial thickening. | Endometrial thickening plus left ovarian cyst (NMR) | OHT plus ovarian cyst plus endometrial adenocarcinoma |
| Bilateral hypoechoic ovarian lesions (11 & 7 mm) | Not performed | OHT |
| Unilateral hyperechoic ovarian imaging (7 mm). Endometrial thickening. | Left adrenal adenoma 24 mm (NMR) | Gonadotropin-dependent hyperandrogenism |
| Solid left ovary with ↑ size (30 * 19 cm) | Not performed | Fibrothecoma |
| Unilateral ovarian tumor with ↑ vascularization (19 * 15 mm) | Not performed | Leydig cell tumor |
| Unilateral hypoechoic ovarian lesion (17 mm) | No abnormal findings (CT) | Granulosa cell tumor |
| Solid left ovary with ↑ size (48 * 42 mm) and central vascularization | No abnormal findings (CT) | Steroid cell tumor-NOS |
| Right ovary (25 mm) including a hyperechoic image (10 mm) and left ovary with an anechoic image (22 mm) | No abnormal findings (CT) | Gonadotropin-dependent hyperandrogenism |
| No abnormal findings | Left ovarian solid and cystic mixed lesion (CT) | Steroid cell tumor-NOS |
| No adnexal findings. Endometrial thickening. | Suspicious left ovarian tumor (15 mm) with ↓ T1 & T2 signal and lesion enhancement with gadolinium (NMR) | Leydig cell tumor |

All individuals of our series received a TV-US procedure except for a patient with NCCAH, another one with a history of bilateral oophorectomy because of endometriosis and a final diagnosis of ACTH-dependent functional hyperandrogenism, and two patients diagnosed with Cushing’s disease and adrenal carcinoma, respectively. An abnormal endometrial thickness was found in 28% and 46% of patients with ovarian or adrenal hyperandrogenism, respectively (χ^2^:1.393, *P* = 0.298). Endometrial thickness was normal in all patients with potentially malignant ovarian tumors. However, it was increased in a patient suffering simultaneously endometrial carcinoma and OHT, and in another patient who had endometrial atypical hyperplasia together with hyperandrogenism that was solved by oophorectomy, although histopathology of both gonads failed to reveal the ovarian source of androgen excess.

*Abbreviations* CT, computerized tomography; NMR, nuclear magnetic resonance; OHT, ovarian hyperthecosis; TV-US, transvaginal ultrasonography.

**Supplementary Table 2.** Dynamic functional tests.

| **Ovarian**  **imaging** | **Adrenal**  **imaging** | **Triptorelin/Cetrorelix**  (Change in total T, %) | **Liddle’s test**  (Change in total T, %) | **Final diagnosis** |
| --- | --- | --- | --- | --- |
| Negative | Positive | ↓73% | - | Adrenal adenoma with aberrant LH/hCG receptors |
| Negative | Positive | ↓17% | - | Bilateral macronodular adrenocortical disease  (resolution of hyperandrogenism after unilateral adrenalectomy) |
| - | Negative | 0% | ↓100% | Functional ACTH-dependent hyperandrogenism |
| Negative | Negative | ↑6% | ↓68% | Functional ACTH-dependent hyperandrogenism |
| Negative | Negative | ↓10% | ↓44% | Functional ACTH-dependent hyperandrogenism |
| Negative | Positive | ↑40% | ↓100% | Functional ACTH-dependent hyperandrogenism |
| Positive | Negative | ↓3% | ↓78% | Functional ACTH-dependent hyperandrogenism |
| Negative | Negative | ↓91% | - | Gonadotropin-dependent hyperandrogenism |
| Negative | Negative | ↓60% | - | Gonadotropin-dependent hyperandrogenism |
| Negative | Positive | ↓52% | 0 | Gonadotropin-dependent hyperandrogenism |
| Negative | Positive | ↓75% | ↑36% | Gonadotropin-dependent hyperandrogenism |
| Negative | Negative | ↓75% | - | Gonadotropin-dependent hyperandrogenism |
| Positive | Negative | ↓76% | - | Gonadotropin-dependent hyperandrogenism |
| Positive | Positive | ↓63% |  | Gonadotropin-dependent hyperandrogenism |
| Positive | Negative | ↓96% | - | Granulosa cell-tumor |
| Negative | Positive | ↓74% | - | Leydig cell hyperplasia |
| Negative | Positive | ↓46% | - | Leydig cell hyperplasia |
| Positive | Negative | ↓2% | - | Mucinous cystadenoma |
| Positive | Positive | 0% | - | Non-classic congenital adrenal hyperplasia |
| Negative | Negative | ↓65% | - | Normal ovarian histology (resolution of hyperandrogenism after oophorectomy) |
| Negative | - | ↓90% | - | Ovarian hyperthecosis |
| Negative | Negative | ↓87% | - | Ovarian hyperthecosis |
| Positive | - | ↓72% |  | Ovarian hyperthecosis |
| Negative | Negative | ↓76% | - | Ovarian hyperthecosis |
| Negative | - | ↓71% | - | Ovarian hyperthecosis |
| Negative | Negative | ↓73% | ↓4% | Ovarian hyperthecosis and non-classic congenital adrenal hyperplasia |
| Positive | Negative | ↓89% | - | Steroid cell tumor-NOS |
| Negative | Positive | ↓50% | ↓35% | Unclear source |

Two patients finally diagnosed with borderline/malignant ovarian tumors showed a decrease in total T after triptorelin administration of 96% and 89%, respectively. In another seven patients, pharmacological gonadotropin-suppression resulted into a ≥ 50% decrease in circulating total T; they were diagnosed with gonadotropin-dependent hyperandrogenism, albeit definite histopathologic diagnosis was lacking. However, none of them developed a clinically apparent androgen-secreting neoplasm during their follow-up (37 ± 14 months). None of those seven individuals diagnosed of functional ACTH-dependent hyperandrogenism developed a clinically apparent androgen-secreting neoplasm during a mean follow-up of 57 ± 28 months.

**References**

1. Ankarberg-Lindgren C & Norjavaara E. Sensitive RIA measures testosterone concentrations in prepubertal and pubertal children comparable to tandem mass spectrometry. *Scand J Clin Lab Invest* 2015 **75** 341-344.

2. Vermeulen A, Verdonck L & Kaufman JM. A critical evaluation of simple methods for the estimation of free testosterone in serum. *J Clin Endocrinol Metab* 1999 **84** 3666-3672.
